# Cancer-Therapy-Related Cardiac Dysfunction: Latest Advances in Prevention and Treatment

**DOI:** 10.3390/life15030471

**Published:** 2025-03-15

**Authors:** Carla Contaldi, Carmine D’Aniello, Domenico Panico, Andrea Zito, Paolo Calabrò, Emilio Di Lorenzo, Paolo Golino, Vincenzo Montesarchio

**Affiliations:** 1 Department of Cardiology, AORN dei Colli-Monaldi Hospital, 80131 Naples, Italy; 2 Division of Medical Oncology, AORN dei Colli-Monaldi Hospital, 80131 Naples, Italy; 3 Department of Translational Medical Sciences, University of Campania “Luigi Vanvitelli”, 80131 Naples, Italy

**Keywords:** cancer-therapy-related cardiac dysfunction (CTRCD), biomarkers, imaging techniques, pharmacological interventions, emerging technologies

## Abstract

The increasing efficacy of cancer therapies has significantly improved survival rates, but it has also highlighted the prevalence of cancer-therapy-related cardiac dysfunction (CTRCD). This review provides a comprehensive overview of the identification, monitoring, and management of CTRCD, a condition resulting from several treatments, such as anthracyclines, HER2-targeted therapies, target therapies, and radiotherapy. The paper includes a discussion of the mechanisms of CTRCD associated with various cancer treatments. Early detection through serum biomarkers and advanced imaging techniques is crucial for effective monitoring and risk stratification. Preventive strategies include pharmacological interventions such as ACE inhibitors/angiotensin receptor blockers, beta-blockers, and statins. Additionally, novel agents like sacubitril/valsartan, sodium-glucose co-transporter type 2 inhibitors, and vericiguat show promise in managing left ventricular dysfunction. Lifestyle modifications, including structured exercise programs and optimized nutritional strategies, further contribute to cardioprotection. The latest treatments for both asymptomatic and symptomatic CTRCD across its various stages are also discussed. Emerging technologies, including genomics, artificial intelligence, novel biomarkers, and gene therapy, are paving the way for personalized approaches to CTRCD prevention and treatment. These advancements hold great promise for improving long-term outcomes in cancer patients by minimizing cardiovascular complications.

## 1. Introduction

The rapidly changing field of cancer treatment has led to remarkable progress, significantly enhancing patient survival outcomes. Nevertheless, the cardiovascular consequences of numerous cancer therapies have become more apparent, revealing a spectrum of cardiotoxic effects. This underscores the critical need for robust strategies in monitoring and management [1,2]. Among the most frequent and severe cardiovascular complications associated with cancer therapies are the onset of left ventricular (LV) dysfunction and heart failure (HF). However, the underlying mechanisms of cardiotoxicity vary across different therapeutic approaches [3,4,5,6,7,8].

Cancer-therapy-related cardiovascular disease (CTRCD) includes cardiac injury, cardiomyopathy, and HF, presenting as either symptomatic or asymptomatic [9]. Asymptomatic CTRCD is categorized as mild (LVEF ≥ 50% with ≥15% relative global longitudinal strain (GLS) decline and/or biomarker elevation), moderate (LVEF decrease ≥ 10% to 40–49% or <10% reduction to same range, plus >15% relative GLS decline or elevated biomarkers), or severe (LVEF < 40%). Symptomatic CTRCD ranges from mild (no therapy change) to very severe (transplant consideration) [5,6,7,8,9].

The American College of Cardiology defines “Definite CTRCD” as a reduction in LVEF of ≥10% but <50%, and “Possible CTRCD” as a reduction in LVEF of ≥10% but between 50 and 55%, a reduction of <10% with LVEF < 50%, or a relative decline in GLS of ≥15% without significant LVEF reduction [10].

CTRCD can occur during or after cancer therapy. Risk assessment, using the Heart Failure Association/International Cardio-Oncology Society (HFA/ICOS) score [7], is recommended before potentially cardiotoxic treatments [5,6,7,8,9]. The current guidelines lack a strong empirical basis, making a universal practical approach challenging.

This review aims to provide a comprehensive and updated understanding of CTRCD, with a particular focus on recent advances and emerging strategies in early detection and cardioprotection. It begins by outlining the main classes of drugs responsible for CTRCD and their underlying mechanisms, shedding light on how these mechanisms can be targeted for early identification and intervention.

This paper not only synthesizes current therapeutic strategies based on the latest ESC guidelines, but also highlights novel cardioprotective approaches, exploring their mechanisms and the evolving evidence from both preclinical and clinical studies.

A key feature of this review is its focus on future research opportunities, particularly the exploration of the pathophysiological mechanisms of cardiotoxicity and the role of cutting-edge technologies in improving prevention and treatment. By doing so, this review aims to encourage further investigation into personalized therapies and innovative techniques that could enhance patient outcomes in the context of CTRCD.

## 2. Classes of Cancer Therapy and Their Mechanisms Causing CTRCD

The cancer treatments most frequently associated with LV dysfunction are anthracyclines, anti-HER-2 therapy, and targeted anticancer therapies, such as Vascular endothelial growth factor inhibitors (VEGF inhibitors), RAF inhibitors, MEK inhibitors, proteasome inhibitors, BCR-ABL inhibitors (dasatinib, ponatinib), and EGFR/HER2 (osimertinib), as well as radiotherapy [9].

*Anthracyclines:* The drugs commonly used in cancer treatment inhibit topoisomerase IIβ, which disrupts DNA repair and leads to cardiac cell necrosis, apoptosis, and irreversible damage [2,4]. Anthracyclines also induce mitochondrial damage [2,4] and generate reactive oxygen species (ROS) [11,12,13,14] and ferric cations (oxidative stress and ferroptosis) [12] that cause DNA damage and disrupt calcium homeostasis (titin degradation), which leads to systolic LV dysfunction [2,4,11,12,13,14].

In addition, anthracyclines cause cardiac inflammation through oxidative damage, triggering immunogenic cell death like pyroptosis and necrosis. This leads to damage-associated molecular patterns (DAMPs) [11], the activation of Toll-like receptors (TLRs) [11,12], and the production of pro-inflammatory cytokines, exacerbating myocardial damage and cardiotoxicity [11]. Furthermore, doxorubicin inhibits natural anti-inflammatory mechanisms in the myocardium, worsening inflammation and contributing to cardiotoxicity [11,12,14]. Inflammation impairs cardiac function not only through cell death, but also by affecting the function of surviving cells, similar to the mechanisms observed in sepsis-induced cardiomyopathy and CAR T-cell therapy-related cardiotoxicity [11,14]. Anthracycline-induced cardiotoxicity is classified as acute (within 2 weeks), early (within 1 year), and late (after 1 year), exhibiting dose-dependent effects (early and late) and variable reversibility [2]. Anthracycline-induced CTRCD is progressive and influenced by cumulative dose. Cumulative doxorubicin ≥ 250 mg/m^2^ or equivalent is considered high risk [9].

*HER2-targeted therapy:* Monoclonal antibodies, crucial in HER2-positive breast cancer (early and metastatic), can induce cardiac dysfunction by disrupting HER-2/ERBB2-neuregulin (NRG) signaling, essential for cardiomyocyte growth, repair, and homeostasis. Trastuzumab binds to HER2, which blocks NRG-1 activation, disabling cardioprotective signaling and increasing vulnerability to reactive oxygen species (ROS), especially with anthracycline co-administration. The HER2 blockade also elevates angiotensin II, further amplifying oxidative stress [2,4,9,11,12,13,14]. Cardiotoxicity typically manifests during therapy, but is reversible, and can lead to LV dysfunction in 15–20% of patients [2,9].

*VEGF inhibitors*: Monoclonal antibodies and tyrosine kinase inhibitors (TKIs) target the VEGF pathway implicated in various cancers. Hypertension is a common adverse event, but HF/LV dysfunction can also occur, due to increased oxidative stress and mitochondrial dysfunction [3,9,13,14].

*TKIs targeting BCR-ABL:* Small-molecule TKIs targeting BCR-ABL have proven to be effective in chronic myeloid leukemia treatment. These drugs can cause LV dysfunction through several interconnected mechanisms. Severe mitochondrial impairment disrupts energy production in cardiomyocytes, reducing the ATP levels essential for heart contraction. This leads to oxidative stress and the activation of cell death pathways, contributing to HF. The inhibition of enzymes like ABL and ARG also disrupts cardiomyocyte function, affecting myocardial performance and vascular tone. These combined effects on mitochondrial function, ATP production, and enzyme activity can result in CTRCD, with varying frequency and severity, depending on the specific drug used [3,9,14].

*Proteosome Inhibitors (PI):* The proteasome plays a key role in degrading abnormal proteins that are tagged with ubiquitin. In cancer cells, this process is often overactive. The anticancer action of this drug class involves inhibiting the proteasome by targeting one of its subunits. The proposed mechanism of cardiotoxicity involves oxidative stress affecting cardiomyocytes, potentially leading to cell death or transient endothelial dysfunction. Pre-existing cardiovascular conditions and the concurrent use of anthracyclines are identified as risk factors for developing cardiac dysfunction [3,9,13,14].

*B-RAF Inhibitors:* The Ras-Raf-MAP kinase pathway is a key growth factor signaling cascade closely associated with cancer development. Ras, a central oncogene, is among the most frequently mutated genes in human cancers. To target this pathway, multiple drugs have been developed to inhibit the kinases activated within it, with some treatments administered in combination for enhanced efficacy. When they are used in combination with MEK inhibitors, there is a higher incidence of HF/LV dysfunction. Cardiotoxicity associated with BRAF/MEK inhibitors is likely due to the disruption of the MAPK signaling pathway within the cardiovascular system. The activation of ERK (extracellular-signal-regulated kinases) plays a crucial role in preserving heart health. ERK1/2 provides several protective functions for cardiomyocytes, including defense against oxidative stress, the prevention of hypertrophy, and the inhibition of apoptosis [3,9,14].

*MEK Inhibitors*: These drugs are part of the effort to inhibit the Ras-Raf-MAP kinase pathway. They inhibit the downstream target of BRAF. LV dysfunction is the main cardiovascular toxicity, particularly in combination therapy with a B-RAF inhibitor. Interruption and/or dose reduction can be associated with the recovery of LV function [3,9,14].

*Immune checkpoint inhibitors (ICIs)*: These are a class of monoclonal antibodies designed to target specific immune checkpoint proteins found on T cells. These proteins normally regulate immune responses, ensuring that T cells do not attack healthy cells. By blocking these checkpoints, ICIs enhance the immune system’s ability to identify and eliminate cancer cells. The main targets of ICIs include cytotoxic T-lymphocyte-associated antigen-4 (CTLA-4), programmed death-1 (PD-1), and programmed death-ligand 1 (PD-L1) [14].

By interfering with these regulatory pathways, ICIs stimulate a more robust T-cell-mediated immune response against cancer cells. However, this heightened immune activity can also cause unintended damage to healthy tissues, leading to immune-related adverse effects. In the cardiovascular system, these effects can present as severe myocarditis, which is the most common and life-threatening complication, with an approximate mortality rate of 50%, as well as myopericarditis and HF. These cardiovascular side effects tend to arise early during treatment. Nevertheless, they can occasionally appear later in the course of treatment [2,3,9,14].

*Radiotherapy (RT):* This option is widely used in cancer treatment and often forms an integral part of a multimodal therapeutic strategy. Its efficacy stems from its ability to cause DNA damage, trigger localized inflammation, and induce tissue fibrosis. Despite its therapeutic benefits, RT can have harmful cardiovascular effects. Repeated ischemic events caused by inflammation may lead to fibrosis of microvascular endothelial cells in the pericardium. Additionally, RT can cause intravascular inflammation in epicardial coronary arteries, impairing oxygen and nutrient delivery to the myocardium and resulting in cardiomyocyte death and fibrosis. RT also fosters a pro-thrombotic state, induces vasospasms, and promotes the recruitment of monocytes and macrophages to the arterial intima, which, along with increased lipoprotein infiltration, accelerates the progression of pre-existing atherosclerosis [2,3,9,13,15]. One proposed mechanism of RT-induced cardiotoxicity is mitochondrial damage, coupled with the activation of NADPH oxidase, leading to the generation of ROS that exacerbate mitochondrial dysfunction [2,15].

These adverse effects may not become apparent until years after completing RT. Factors that increase the risk of cardiotoxicity include higher radiation doses (≥30 Gy), concurrent use of anthracyclines, direct irradiation of the heart, younger age (<25 years), pre-existing cardiovascular conditions, and other cardiovascular risk factors [9].

**Figure 1:**
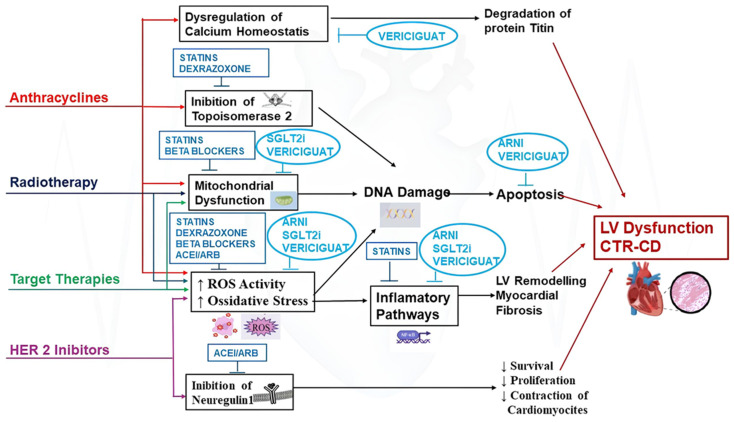
Main mechanisms of cardiotoxicity associated with anthracyclines, radiotherapy, targeted therapies, and HER2 inhibitors; and the possible protective mechanisms of statins, dexrazoxane, beta-blockers, ACEI/ARB, and the novel sacubitril/valsartan (ARNI), sodium-glucose co-transporter type 2 inhibitors (SGLT2i), and vericiguat in preventing LV systolic dysfunction (CTRCD). In the blue rectangles, the drugs recommended by the ESC cardioncology guidelines are listed; in the light blue circles, the novel cardioprotective agents are listed.

Figure 1 summarizes the main mechanisms of cardiotoxicity associated with anthracyclines, radiotherapy, targeted therapies, and HER2 inhibitors, as well as the potential mechanisms of cardioprotective drugs. Table 1 provides a summary of cancer treatments that can cause CTRCD, their oncologic indication, their possible mechanism of cardiotoxicity, and the frequency with which they may lead to LV dysfunction.

## 3. Early Detection and Monitoring of CTRCD

### 3.1. Serum Cardiac Biomarkers

Research on using serum cardiac biomarkers to predict the risk of cardiotoxicity prior to cancer treatment remains limited, with current guidelines largely relying on expert opinion. The 2022 ESC guidelines recommend the baseline measurement of cardiac biomarkers such as cardiac troponins (cTnI or cTnT) and natriuretic peptides (NPs)—including B-type natriuretic peptide (BNP) and N-terminal pro-BNP (NT-proBNP)—to assess cardiovascular risk in patients undergoing therapies like anthracyclines, HER2-targeted treatments, and VEGF inhibitors [9]. Monitoring these biomarkers during therapy can help to identify patients who may benefit from cardioprotective interventions. The baseline levels of NPs and/or cTn are especially recommended for patients at elevated risk of CTRCD when biomarker fluctuations are used for tracking over the course of treatment. However, universally accepted cutoff values for these biomarkers in cancer patients are lacking, as their levels can be influenced by age, renal function, and other comorbidities. Consequently, biomarker changes should be interpreted in the broader context of each patient’s clinical profile and treatment timeline [8,9]. For instance, an elevation in troponin levels following anthracycline therapy has been strongly associated with an increased risk of LV dysfunction. A 2020 meta-analysis reported that troponin elevation after treatment increased the risk of LV dysfunction seven-fold, with a high negative predictive value of 93%. While BNP and NT-proBNP levels also rise after chemotherapy, evidence regarding their predictive accuracy for LV dysfunction is less consistent [16].

### 3.2. Imaging Techniques for Monitoring Cardiac Function

Cardiovascular imaging plays a pivotal role in identifying the early signs of cardiac dysfunction, evaluating pre-existing heart conditions prior to initiating cancer therapy and establishing a baseline for tracking changes throughout and after treatment [9].

A baseline assessment of LV function is critical within the three months before beginning potentially cardiotoxic cancer treatments to detect any underlying cardiac abnormalities.

*Transthoracic echocardiography* is the cornerstone of LV dysfunction diagnosis, with LVEF as a primary metric [9]. Three-dimensional echocardiography is preferred for LVEF and cardiac volume evaluation due to superior accuracy and lower interobserver variability (5–6%) [9,17]. If 3D imaging is unavailable, the modified biplane Simpson’s method is recommended [9,18]. Ultrasound contrast agents can improve LV function and volume assessment when image quality is suboptimal [9,19]. GLS, derived from speckle tracking, is a more sensitive and reproducible measure of LV systolic function than LVEF, detecting subclinical dysfunction before LVEF changes [20,21,22]. It is crucial for pre-treatment risk stratification and should be included in baseline evaluation [20]. GLS < −16% indicates abnormal function, >−18% normal, and −16% to −18% borderline. Both LVEF and GLS are essential for cardiotoxicity monitoring [20,21,22,23,24]. A ≥15% GLS reduction from the baseline suggests subclinical cardiotoxicity and an increased risk of future LV dysfunction. GLS measurements can vary slightly (up to 3.7%) depending on the equipment used. Chemotherapy-induced loading condition changes can affect GLS readings. Blood pressure measurement during resting echocardiography is recommended [9,25]. Myocardial work (global work index, global constructive work, global wasted work, and global work efficiency) integrates myocardial deformation and afterload, showing promise as an early cardiotoxicity marker, particularly with anthracycline or anthracycline/trastuzumab therapy, but requires further research [26,27,28,29,30].

Right ventricular (RV) abnormalities (e.g., reduced tricuspid annular plane systolic excursion or RV free wall systolic peak S’) have prognostic significance in anthracycline/trastuzumab-treated patients [31]. RV free wall longitudinal strain is a potential early marker of subclinical RV toxicity, but needs further validation [32,33].

Diastolic function decline (e’, E/e’ avg ratio) is observed in anthracycline-treated patients and often precedes systolic impairment, but its prognostic relevance is limited [34].

Left atrial reservoir strain is a potential marker for subclinical cardiotoxicity in these patients [35].

Stress echocardiography, preferably exercise-based, can evaluate subclinical LV and RV dysfunction and contractile reserve [36,37,38]. Pharmacologic stress can be used when exercise is not feasible. A reduced contractile reserve (at least a 5% reduction) on *dobutamine stress echocardiography* in patients treated with anthracyclines identifies patients at high risk developing late LV systolic dysfunction early [39,40]. However, its application in the early detection of cardiotoxicity is limited due to a lack of robust supporting evidence in the literature [40,41]. Reduced coronary flow reserve (CFR) in the left anterior descending (LAD) coronary artery during *dipyridamole stress echocardiography* has been observed in patients treated with anticancer drugs that cause direct endothelial damage, such as 5-Fluorouracil, or endothelial dysfunction through nitric oxide inhibition, like VEGF inhibitors (e.g., bevacizumab and sunitinib). In particular, in patients treated with sunitinib, reduced CFR in the LAD coronary artery is an early marker of cardiotoxicity and correlates inversely with treatment duration and inflammation markers [36,42].

New echocardiographic parameters, such as *hemodynamic forces*, which are used to assess LV dysfunction and predict cardiac remodeling, could serve as a novel early marker of subclinical cardiotoxicity [43,44].

When transthoracic echocardiography produces low-quality images, *cardiac magnetic resonance (CMR)* is an effective alternative for detecting CTRCD, due to its accuracy, reliability, and sensitivity to subtle changes in both LV and RV function. Additionally, CMR allows tissue characterization that can help to identify the etiology of LV systolic dysfunction. CMR temporal variability for LVEF measurements is low (approximately 2.4–7.3%) [45]. Functional changes in RV following chemotherapy often coincide with declines in LVEF, especially in patients receiving combined treatments with trastuzumab [46].

Recent studies show that *CMR-derived GLS,* using feature tracking (FT), can identify LV dysfunction before LVEF decline and predict overall mortality across different cardiomyopathies [47]. Reductions in global circumferential and longitudinal strain are observed in patients treated with doxorubicin and trastuzumab, correlating with subclinical LVEF decreases, highlighting its role in early cardiotoxicity monitoring [48,49]. However, FT-CMR has higher variability than echo-based GLS, and more data are needed to establish its effectiveness in managing CTRCD outcomes [50].

Anthracycline therapy reduces *LV mass,* measured by CMR, which can serve as a marker of cardiotoxicity. This reduction is due to cardiomyocyte shrinkage (up to 40%) with partial compensation by increased extracellular volume (ECV) [51]. Furthermore, CMR studies have demonstrated the presence of early myocardial edema after anthracycline therapy, detectable through T2-weighted imaging sequences [52,53]. Although myocardial fibrosis is a common anthracycline side effect, it is rarely detected with LGE because anthracycline-related fibrosis tends to be diffuse and interstitial, which limits LGE’s visualization capacity [52,53,54]. Diffuse fibrosis may become apparent years post-treatment and can be evaluated with T1 mapping [55,56]. CMR can distinguish cardiotoxicity characterized by early inflammation (elevated native T1 and T2 values) from that characterized by later cardiac remodeling due to interstitial fibrosis (increased native T1 values only) [57,58]. ECV, which increases in patients following anthracycline therapy, is associated with diastolic dysfunction, larger atrial volumes, and increased short-term mortality [59]. ECV rises not only due to interstitial expansion, but also due to cardiomyocyte loss and atrophy. [60,61]. Both *native T1* and *ECV* seem to be early biomarkers of CTRCD, able to detect subclinical myocardial dysfunction.

*Multigated acquisition nuclear imaging (MUGA)* is now considered a third-line option for assessing LVEF when echocardiography and CMR are unavailable. Its use is limited due to radiation exposure and its inability to assess RV function, atrial size, or valvular and pericardial conditions. MUGA is now recommended only in specific cases—such as in patients with poor echocardiographic windows, those with implanted devices incompatible with CMR (e.g., post-mastectomy patients with tissue expanders), or in facilities lacking access to CMR [9,11].

*Cardiac computed tomography (CCT)* can effectively assess heart chamber function, offering detailed volumetric and structural data for both ventricles through retrospective multiphase ECG-gated imaging. This makes CCT a reliable alternative to CMR for functional and morphological analysis [62,63]. Additionally, in patients with a low to intermediate pre-test probability of coronary artery disease (CAD), CCT provides high sensitivity in ruling out obstructive CAD (high negative predictive value) [64,65]. Recently, quantification of coronary artery calcium scoring with non-contrast cardiac CT has been proposed for assessing cardiovascular risk in all cancer patients and cancer survivors [66]. Emerging evidence also suggests that *CCT-derived ECV* may serve as a useful biomarker for tracking anthracycline-induced cardiotoxicity in breast cancer patients [67,68].

## 4. Pharmacological Interventions for Prevention of CTRCD

According to the 2022 ESC cardio-oncology guidelines, for the primary prevention of cardiotoxicity in high- or very-high-risk patients (as assessed by the HFA-ICOS score) undergoing anthracycline and/or anti-HER-2 therapy or other targeted anticancer therapies that may induce LV systolic dysfunction, *ACE inhibitors (ACEI) or angiotensin receptor blockers (ARB)*, along with *beta-blockers*, are recommended (class IIa) [9].

*ACEIs* and *ARBs* attenuate oxidative stress and also improve mitochondrial function, cardiomyocyte metabolism, and intracellular calcium handling. From preclinical and clinical studies, it appears that anthracyclines lead the dysregulation of the renin–angiotensin–aldosterone system (increased levels of angiotensin II, overexpression of angiotensin type 1 receptor, and decreased expression of MasR and AT2R receptors), which contributes to anthracycline-related cardiotoxicity. It appears that the increased cardiac stress resulting from the HER-2 blockade leads to the upregulation of angiotensin II, which causes increased oxidative stress through NADPH oxidase activation [69,70,71,72,73,74] . Additionally, some studies suggest that angiotensin II may directly reduce NRG-1 levels [75,76]. Consequently, elevated angiotensin II levels can exacerbate cardiomyocyte damage during trastuzumab treatment [77]. This mechanism may explain the potential benefit of renin–angiotensin–aldosterone system antagonists in managing HER2 inhibitor cardiotoxicity. The cardioprotective effects of ACEIs have been documented in both retrospective and prospective studies, showing a reduction in the risk of LVEF decline in patients treated with doxorubicin who also used ACEIs. The timely initiation of ACEI therapy, ideally within six months of chemotherapy, is associated with better outcomes [78,79]. Prospective studies, including a randomized trial, have demonstrated that enalapril plays a role in preventing LVEF decline [80,81,82]. A randomized trial showed that enalapril prevented LVEF decline and troponin elevation in patients receiving doxorubicin, mitigating both systolic and diastolic dysfunction [80]. Cardinale et al. found that enalapril prevented significant LVEF decline in patients with elevated troponin I post-anthracycline chemotherapy, also preventing increases in cardiac volumes and drastically reducing cardiac event risk [81]. The ICOS-ONE trial, comparing universal vs. troponin-triggered enalapril initiation, found no significant difference in troponin elevation, suggesting routine ACEI use despite troponin’s inconsistent correlation with cardiotoxicity [82]. Other ACEIs have shown promise in smaller studies. A single-arm trial involving ramipril and/or bisoprolol in 35 high-risk non-Hodgkin lymphoma patients demonstrated a lower incidence of cardiac damage, preserved LVEF, and improved survival compared to historical controls. However, the baseline differences between groups limit direct comparisons [83]. Another study evaluating perindopril in 68 patients treated with epirubicin found better preservation of LVEF, though diastolic dysfunction and QTc prolongation persisted [84]. In addition, *ARBs*, such as valsartan, telmisartan, and candesartan, have shown cardioprotective effects. Nakamae et al. conducted a pilot study on 40 non-Hodgkin lymphoma patients. The patients were randomized to receive *valsartan* (80 mg/day) or a placebo during chemotherapy. While valsartan significantly reduced BNP levels and prevented LV dilation and QTc interval prolongation, no impact on LVEF was observed due to the short observation period (7 days) [85]. Cadeddu et al. studied *telmisartan* (40 mg/day) in 49 epirubicin-treated patients, administered 1 week before and throughout chemotherapy. While LVEF remained unchanged, telmisartan prevented LV diastolic dysfunction, as shown by preservation of the early/late diastolic peak velocity (E/A) ratio. It also mitigated reductions in strain rate peak, an early indicator of myocardial dysfunction, after repeated chemotherapy doses. Additionally, telmisartan reduced ROS and IL-6, suggesting anti-inflammatory and antioxidant benefits. Despite these findings, the small sample size and short follow-up limited the study’s broader applicability [86,87]. The PRADA trial explored the use of candesartan in preventing anthracycline-induced cardiotoxicity, showing a reduction in LVEF decline, although further long-term studies are needed to confirm these results [88].

*Beta-blockers* possess cardioprotective properties, including antioxidant and anti-apoptotic effects [89,90,91,92,93,94]. Clinical studies, including the CarDHA trial and others, have shown that carvedilol reduces doxorubicin-induced cardiotoxicity, preserving LVEF and improving cardiac function. However, it does not always significantly reduce HF hospitalizations or cardiac deaths [95,96,97,98,99,100]. The OVERCOME trial demonstrated that enalapril and carvedilol reduced the incidence of severe cardiac events and LVEF decline in patients with hematologic malignancies undergoing intensive chemotherapy [101].

Additionally, *spironolactone,* an aldosterone receptor antagonist that inhibits the final step of the renin–angiotensin–aldosterone system, has demonstrated antioxidative effects and has been explored for its potential benefits, although research in this area remains limited [102,103].

However, neurohormonal blockers, while effective in managing LV dysfunction, do not seem to be sufficiently effective in preventing drug-induced ventricular dysfunction caused by cancer therapies, which typically trigger only moderate neurohormonal responses. Randomized clinical trials involving low- to moderate-risk patients treated with anthracyclines have not demonstrated significant cardioprotective benefits from carvedilol (CECCY trial) [98], candesartan (PRADA trial) [88], or a combination of candesartan and carvedilol (CardiacCARE trial) [104]. However, an interim analysis of a study on breast cancer patients showed that combined therapy with ramipril and bisoprolol can prevent LVEF decline even in low-risk patients [105].

Finally, the ESC guidelines recommend the use of dexrazoxane and liposomal anthracyclines in patients at high or very high risk of cardiotoxicity who require anthracycline therapy [9].

*Dexrazoxane* is an iron-chelating agent with a cardioprotective effect during anthracycline treatment. It reduces intracellular iron accumulation, which leads to increased ROS and inhibits the formation of topoisomerase-anthracycline complexes. Both mechanisms result in reduced apoptosis, necroptosis, and less LV remodeling [106]. A recent meta-analysis by Keshavarzian et al. supported the efficacy of cardio-protective drugs, including dexrazoxane, beta-blockers (carvedilol and bisoprolol), and ACEI drugs, in patients undergoing chemotherapy with anthracycline, which have a protective effect on LVEF and prevent EF drop [107].

Moreover, *liposomal anthracyclines*, due to their different pharmacokinetics and tissue distribution, offer equivalent antitumor efficacy with reduced cardiotoxicity.

Additionally, *statins* are recommended (class IIa) for all adult cancer patients at high or very high risk of cardiotoxicity [9]. The possible mechanisms of cardioprotection provided by statins, beyond cholesterol, reduction stabilize atherosclerotic plaques and decrease cardiovascular morbidity and mortality [108], are due to their pleiotropic effects, such as reducing oxidative stress, anti-inflammatory effects, antifibrotic activity, endothelial protection, and apoptosis reduction. Moreover, statins have immunomodulatory effects, such as reducing the expression of major histocompatibility complex class II, which dampens the immune response by decreasing T cell proliferation and regulating T cell differentiation. They also limit leukocyte recruitment by inhibiting chemokine secretion and ICAM-1 expression [109]. Many preclinical studies have evaluated the protective mechanisms of statins in preventing cardiotoxicity induced by anthracyclines (doxorubicin) [110,111,112], cyclophosphamide [113], trastuzumab [114], the combination of trastuzumab and doxorubicin [115], 5-FU [116], radiotherapy [117,118,119], and ICIs like pembrolizumab [120].

More recently, several clinical studies have assessed the cardioprotective role of statins in patients treated with anthracyclines [121,122,123,124,125]. A recent meta-analysis of three observational studies and four randomized controlled trials (RCTs) found a reduced incidence of cardiotoxicity in patients treated with statins, although there was no significant change in LVEF between the intervention and control groups [126]. In a retrospective case–control study of 525 patients with HER2-positive breast cancer undergoing trastuzumab-based therapy, statin treatment was independently associated with a lower risk of cardiotoxicity [127]. However, given differences in patient populations, statin regimens, and the small sample sizes of studies, further mechanistic studies and large-scale clinical trials are needed. Moreover, it appears that, compared to men, women are at higher risk of non-adherence to statin treatment and are more prone to discontinuing the therapy due to side effects, which are related to a different metabolism of statins (lower glomerular filtration rate, higher body fat percentage, and overall faster statin metabolism). Therefore, optimizing statin treatment in women requires adjusting the doses based on renal function and muscle symptoms. Additionally, in elderly women undergoing chemotherapy, it is crucial to consider potential drug interactions, simplify the treatment regimen, and assess adherence to therapy [108].

Polyphenols have been shown to reduce statin side effects through their antioxidant and anti-inflammatory properties. They seem to protect against muscle damage by reducing oxidative stress and may alleviate statin-induced liver enzyme elevation with their hepatoprotective effects. Additionally, flavonoids, like dihydromyrecetin, have shown potential in reducing doxorubicin-induced cardiotoxicity by modulating inflammation through the nucleotide-binding oligomerization domain-like receptor family pyrin domain containing 3 (NLRP3) inflammasome [11,128,129,130]. However, these effects have currently only been observed in animal and in vitro models; therefore, human studies are needed.

Table 2 summarizes the main clinical trials, and Table 3 provides an overview of the main meta-analyses evaluating pharmacological cardioprotective strategies for the primary prevention of CTRCD.

## 5. Pharmacological Interventions for Treatment of CTRCD

According to the 2022 ESC cardio-oncology guidelines, for the treatment of asymptomatic mild CTRCD caused by anthracyclines and anti-HER2 therapy, the use of ACEIs, ARBs, and/or beta-blockers should be considered without discontinuing oncological therapy, with close cardiac monitoring every one or two chemotherapy cycles. Research has shown that ACEIs (e.g., enalapril) and beta-blockers (e.g., carvedilol or bisoprolol) can effectively reverse this condition [138]. The success of the treatment depends on early intervention; moreover, therapy initiated within two months after chemotherapy showed a 64% complete recovery rate in patients with reduced LVEF (≤45%). Conversely, treatment delays of 6–12 months yielded little to no improvement [139].

Symptomatic CTRCD treatment follows HF guidelines, using ACEIs, ARBs, ARNI (angiotensin Receptor-Neprilysin Inhibitor, sacubitril/valsartan), beta-blockers, sodium-glucose co-transporter type 2 inhibitors (SGLT2i), and mineralocorticoid receptor antagonists (MRA), tailored to LVEF and natriuretic peptide levels [9,139,140,141]. Multidisciplinary discussions help to balance cardiac and oncological needs. Discontinuation of anthracyclines is advised for severe CTRCD and requires reassessment for mild and moderate cases. Liposomal anthracyclines or dexrazoxane may be used to reduce cardiotoxicity when resuming treatment [9]. Regarding anti-HER2 therapy, temporary discontinuation is recommended for moderate-to-severe CTRCD, with reassessment for resumption. For mild cases, a multidisciplinary approach is also advised. In severe asymptomatic CTRCD caused by anti-HER2 therapy, HF treatment and temporary discontinuation are recommended, with reassessment for therapy resumption [9].

Although the ESC HF guidelines [140] recommend ARNI for patients with HF and reduced LVEF in class I, there is still little evidence of its efficacy and safety in CTRCD. Historic results on the use of sacubitril/valsartan in this setting of patients were presented in a Spanish retrospective multicenter registry [142]. This study demonstrated that sacubitril/valsartan promotes reverse LV remodeling and functional recovery, improving LVEF (33% to 42%, *p* ≤ 0.01), reducing pro-BNP levels, and enhancing NYHA class (2.2 ± 0.6 to 1.6 ± 0.6, *p* ≤ 0.01), without significant differences in creatinine and potassium levels. Similar results were observed in a single-center study conducted by Gregoretti et al. [143], which included 635 breast cancer patients. Patients with LV dysfunction and HF were initially treated with ACEIs (enalapril) or ARBs, MRAs, digitalis, and diuretics. Sacubitril/valsartan was introduced in 28 patients who remained symptomatic and met the PARADIGM-HF inclusion criteria. These patients showed a reduction in pro-BNP levels, an improvement in NYHA class (from III-IV to I-II), and an increase in LVEF (from 26.7 ± 5.4% to 32.3 ± 5.5%; *p* < 0.001), without significant differences in creatinine and potassium levels.

*SGLT2is* are increasingly recognized as a key therapeutic option for both HF with reduced EF and HF with preserved EF, owing to their diverse cardioprotective properties [140]; however, there is still limited evidence regarding their effectiveness in the treatment of CTRCD. EMPACARD-treatment is a prospective registry designed to assess the impact of empagliflozin on LV remodeling in patients with metastatic breast cancer and refractory CTRCD. Patients with metastatic breast cancer who experienced refractory CTRCD (characterized as symptomatic HF, despite receiving optimal doses of neurohormonal therapies, including sacubitril/valsartan) during or following treatment with anthracyclines and/or HER2-targeted therapies (such as trastuzumab) were enrolled and administered empagliflozin at a daily dose of 10 mg in addition to their standard HF therapies. HF therapy aligned with clinical guidelines, including empagliflozin, demonstrated in these patients with refractory CTRCD improvements in LV remodeling, with a reduction in pro-BNP levels, improvement in functional class, and an increase in LVEF [144]. The Brazilian cardio-oncology guidelines recommend prescribing SGLT2is to cancer patients who have HF and diabetes mellitus [145].

*Vericiguat*, a new drug with a mechanism of action distinct from the well-known neurohormonal blockade, has shown efficacy in patients with symptomatic HF with reduced EF despite optimal medical therapy (sacubitril/valsartan, beta-blockers, MRAs, and SGLT2is) and a recent episode of worsening HF [140]. A very recent study demonstrated the favorable role of adding vericiguat to standard HF therapy in a young adult cancer survivor who had received high-dose anthracyclines. The patient developed HF with reduced LVEF 20 years after treatment and experienced worsening HF 1.5 years after discharge. Following the addition of vericiguat, the HF symptoms improved, with significant cardiac reverse remodeling, and the patient successfully underwent aortic valve replacement for severe aortic stenosis [146].

## 6. Novel Cardioprotective Drugs for Prevention of CTRCD

### 6.1. Sacubitril/Valsartan

Preclinical studies have shown that sacubitril/valsartan prevents anthracycline-induced CTRCD, not only due to its potent influence on the renin–angiotensin–aldosterone axis, but also by reducing myocardial oxidative stress and inflammation through the regulation of the AMPKα–mTORC1 signaling pathway, mitigating anthracycline-induced apoptosis, and inhibiting autophagy in primary cardiomyocytes [147,148].

The SARAH trial was a recent study presented in November 2024 at the American Heart Association conference. It evaluated the efficacy of sacubitril/valsartan in preventing anthracycline-induced CRTCD. The study involved 114 high-risk patients (with elevated hs-cTnI levels after any dose of anthracyclines, who were mostly women with a mean age of 52) treated at a cancer center in Brazil between March 2022 and August 2024. The participants were randomized to receive either sacubitril/valsartan or a placebo for 6 months, starting with a low dose and titrating to an optimal level. As a primary outcome, treatment with sacubitril/valsartan showed a lower percentage of patients developing a ≥15% reduction in GLS at 6 months, compared to the placebo (7.1% vs. 25.0%, HR 0.23, 95% CI 0.07–0.75; *p* = 0.015), thus reducing the risk of developing subclinical cardiotoxicity [149]. The MAINSTREAM trial [150] focusing on the role of sacubitril/valsartan in the cardioprotection of breast cancer patients is ongoing. Following randomization, the patients will receive treatment with either sacubitril/valsartan or a corresponding placebo for up to 24 months. The primary endpoint of the study has been defined as the occurrence of a decrease in LVEF by ≥5% assessed by echocardiography within 24 months. The estimated completion of the study and publication of the results are expected in December 2027. Future studies focusing on clinical endpoints in patients at even greater risk of cardiotoxicity, such as those with low-normal LVEF or borderline GLS along with elevated hs-cTnI, could be valuable.

### 6.2. SGLT2i

SGLT2is are emerging as promising agents in cardio-oncology, offering potential protection against CTRCD. Preclinical studies, particularly mouse models, have demonstrated that SGLT2is can reduce LV dysfunction caused by anthracyclines. This protective effect is mediated through multiple mechanisms, including enhanced cardiac energy metabolism, reduced oxidative stress, preserved mitochondrial integrity, anti-inflammatory effects, and decreased myocardial fibrosis and cell death [151,152,153,154,155].

Clinical evidence further supports the potential of SGLT2is in cancer patients receiving cardiotoxic therapies. Retrospective observational studies have linked the use of SGLT2is to lower cardiac event rates and improved survival outcomes. One such study, involving cancer patients with diabetes treated with anthracyclines, found that those on SGLT2is experienced fewer HF hospitalizations and less cardiomyopathy. These patients also had better overall survival, with lower rates of sepsis and neutropenic fever compared to the controls [156]. Similarly, another population-based study of older adults (≥65 years) undergoing anthracycline treatment revealed that SGLT2i use was associated with fewer HF-related hospitalizations, although it did not prevent new-onset HF [157].

A larger retrospective analysis of 1280 patients exposed to various cardiotoxic therapies, including anthracyclines and tyrosine kinase inhibitors, demonstrated that SGLT2i users had lower risks of HF exacerbations, all-cause mortality, hospitalizations, atrial fibrillation, acute kidney injury, and the need for renal replacement therapy compared to non-users [158].

The EMPACARD-PILOT trial, a prospective case–control study, specifically evaluated empagliflozin (10 mg/day) in high-cardiotoxic-risk breast cancer patients undergoing anthracycline therapy. The trial found that empagliflozin significantly reduced the incidence of CTRCD (6.5% vs. 35.5%, *p* = 0.005) and preserved LVEF over six months, although no significant differences were observed in biomarkers like NT-proBNP or hospitalization rates [159]. In a recent large-scale study using the TriNetX platform, Bhatti et al. analyzed over 95,000 cancer patients with diabetes and found that SGLT2 inhibitors were associated with a lower risk of CTRCD, with empagliflozin showing the strongest effect. This study suggested broad cardioprotective benefits of SGLT2is across various cancer treatments, including anthracyclines, monoclonal antibodies, and tyrosine kinase inhibitors, highlighting their potential to not only protect the heart, but also enhance cancer treatment outcomes. However, the study’s limitations, such as potential biases and reliance on surrogate endpoints, underscore the need for further research [155].

While these studies demonstrate encouraging results, long-term follow-up and randomized clinical trials are essential to better understand the efficacy and safety of SGLT2is in preventing CTRCD, particularly in cancer patients, both with and without diabetes.

### 6.3. Vericiguat

Vericiguat is a stimulator of soluble guanylate cyclase (sGC), an enzyme that catalyzes the synthesis of cyclic guanosine monophosphate (cGMP), responsible for vasodilatory, antiproliferative, anti-inflammatory, antifibrotic, and proper mitochondrial function effects. In cellular models, vericiguat has shown significant protective effects against doxorubicin-induced apoptosis and inflammation. This drug reduces lactate dehydrogenase release, improves mitochondrial function, and lowers pro-apoptotic markers, helping to preserve heart muscle integrity [160,161]. Vericiguat’s cardioprotective effects are thought to be due to its ability to reduce inflammation by modulating key pathways such as the NLRP3 inflammasome and cytokine production. Additionally, it helps to prevent mitochondrial dysfunction by maintaining calcium balance and supporting mitochondrial biogenesis, which is vital for the survival of heart cells. Furthermore, by increasing cGMP levels, vericiguat promotes vasodilation and reduces afterload, which in turn helps to sustain LVEF. Beyond its cardioprotective properties, vericiguat has also demonstrated potential in preventing sarcopenia, or muscle wasting, which is often induced by chemotherapy [162]. Despite these promising findings, there are limited data on the synergistic effects of vericiguat with other cardioprotective treatments, such as beta-blockers, ACEIs, ARBs, ARNI, or SGLT2is. Further preclinical and clinical studies on the role of vericiguat in preventing CTRCD will be valuable for optimizing therapeutic strategies in patients undergoing cancer treatments.

## 7. Lifestyle Modifications and Non-Pharmacological Strategies for Prevention of CTRCD

### 7.1. Exercise and Rehabilitation

A large amount of evidence from preclinical research, along with increasing clinical findings, indicates that exercise is cardioprotective in settings of anthracycline-associated CTRCD. Mechanisms implicated in exercise-mediated cardioprotection include the modulation of oxidative stress, the protection of mitochondria, and the preservation of cardiac and vascular structure and function, as well as exerkine-mediated effects [163]. Aerobic exercise, plus resistance training, results in better LVEF [164] and better cardiac output [165], increases cardiorespiratory function [166], and reduces cardiovascular morbidity and HF hospitalizations [167]. The BREXIT study demonstrated that a 12-month exercise training program, conducted with 104 women diagnosed with early-stage breast cancer, did not significantly reduce functional disability but did lead to notable improvements in VO2 peak and cardiac reserve [168]. These findings align with a recent meta-analysis of 16 randomized controlled trials (RCTs), which reported enhanced cardiorespiratory fitness and reduced therapy-induced cardiotoxicity among cancer survivors. The benefits were particularly evident with medium- to long-term exercise interventions of moderate-to-high or high intensity, especially when combined with concurrent chemotherapy [169]. Rehabilitation programs, including exercise, are safe and may help to attenuate LVEF decline in women with breast cancer receiving anthracyclines and/or anti-HER2 antibodies and reduce BMI in obese patients [170]. Exercise regimens should be customized based on individual health status, cancer treatment, and cardiovascular risk factors. Randomized clinical trials are currently underway to specifically evaluate the effect of exercise in preventing CTRCD.

### 7.2. Nutritional Interventions

In studies with animal models, calorie restriction has demonstrated a protective role against anthracycline-induced cardiac dysfunction, and, when combined with physical exercise, it shows additive benefits in providing such protection [171].

A recent review including seven studies, six of which focused on oral supplementation strategies with antioxidant and anti-inflammatory properties in breast cancer patients undergoing chemotherapy and one on nutritional counseling and adherence to the Mediterranean diet in breast cancer survivors post-treatment, demonstrated a significantly attenuated reduction in LVEF and significant improvements in blood serum levels of cardiac biomarkers [172]. Nutritional interventions appear to have potential in preventing CTRCD; however, there is currently limited evidence to support this.

## 8. Future Directions and Innovative Technologies for Prevention, Early Detection, and Treatment of CTRCD

### 8.1. Pharmacogenomics and Epigenetics in Predicting CTRCD

The emerging field of pharmacogenomics offers significant potential for predicting individual cardiotoxic risks in oncology patients, enabling personalized therapeutic approaches. Genetic variants that affect drug metabolism and transport have been identified as crucial determinants in the susceptibility to chemotherapy-induced cardiotoxicity.

For instance, variants in ABC genes, which regulate drug export across cell membranes, can impair drug clearance and lead to accumulation in myocardial cells, thereby increasing cardiotoxic risk in some patients. Conversely, variants in soluble carrier transporters (SLC) enhance drug excretion and may reduce the risk of anthracycline-induced cardiotoxicity [173,174,175,176,177]. The CBR3 gene encodes carbonyl reductase-3, an enzyme involved in anthracycline metabolism [178]. Specific variants, such as CBR3:GG, are linked to a higher risk of anthracycline-induced cardiomyopathy, compared to the CBR3:GA/AA genotype (*p* = 0.006) [179]. Similarly, the HAS3 gene, which is responsible for hyaluronic acid synthesis, plays a protective role against oxidative stress and myocardial apoptosis. Variants like rs2232228 influence cardiotoxicity risk at different anthracycline doses. Patients with the AA genotype show lower HAS3 mRNA expression, impaired hyaluronic acid synthesis, and increased cardiotoxic risk [180,181,182]. Additionally, variants such as the Ile654Val SNP in the HER2 gene have been associated with anti-HER2-therapy-induced cardiotoxicity. Breast cancer patients with the Ile/Val genotype experienced a 20% reduction in LVEF during trastuzumab treatment [183]. Epigenetics also plays a pivotal role in chemotherapy-related cardiotoxicity. Circulating methylated DNA from damaged myocytes has been proposed as a potential biomarker for cardiotoxicity. The overexpression of the METTL4 gene, which leads to increased mitochondrial DNA methylation, has been linked to mitochondrial dysfunction and heart failure [184,185]. Bauer et al. demonstrated distinct CpG methylation patterns in peripheral blood mononuclear cells (PBMCs) that corresponded to myocardial functional states before and after doxorubicin treatment. These methylation profiles could serve as predictive markers for chemotherapy-induced cardiotoxicity [186]. Integrating genetic and epigenetic profiling provides a promising strategy to stratify oncology patients based on their cardiotoxicity risk. This approach may facilitate the early detection of cardiac complications, guide timely cardiological interventions, and enable tailored modifications to oncological treatments, ultimately improving patient outcomes.

### 8.2. Artificial Intelligence (AI) for Prediction of CTRCD Risk and Treatment Response

Recent advances in *AI* have established it as a powerful and precise tool for detecting cardiac dysfunction related to anticancer treatments. AI has the potential to improve the reproducibility and accessibility of diagnostic methods used to assess cardiotoxic risk, facilitating more personalized therapeutic approaches. Machine learning models have been developed to predict the risk of CTRCD, taking into account echocardiographic and laboratory parameters [187,188]. A retrospective study [189] explored the correlation between conventional GLS measurements and *AI-based GLS*. In this study, 94 patients receiving chemotherapy were monitored using a software platform called “Caas Qardia”, which incorporated an artificial endocardial border detection algorithm. The results demonstrated a significant correlation (*p* < 0.01) between conventional and AI-based GLS measurements, with the AI method showing greater reproducibility. The AI-based GLS was not significantly affected by cardiac abnormalities or cardiovascular risk factors, which further supports its potential for broader clinical implementation. Another promising AI-based method for risk stratification in cardio-oncology is *AI-enhanced electrocardiography (AI-ECG).* This model predicts the presence and future development of LV systolic dysfunction from ECG images [190]. By calculating a probability score for LV systolic dysfunction, AI-ECG has been shown to correlate with GLS measurements, helping to identify patients at higher risk for cardiac toxicity due to anticancer treatments. In a study by Evangelos et al., AI-ECG was shown to reliably predict LV systolic dysfunction and detect those at a heightened risk for chemotherapy-induced cardiac damage, thus enabling timely treatment adjustments [191]. These AI-driven methods, including GLS and ECG, offer practical, cost-effective alternatives for monitoring oncology patients, irrespective of clinical expertise. By integrating clinical and laboratory data with multi-modal imaging, AI enhances the ability to define individual phenotypes and improve predictions of cardiotoxicity risk and treatment outcomes. However, these models still require validation on large external cohorts and further refinement to reach clinical applicability.

### 8.3. Novel Biomarkers and Molecular Targets for Early Detection of CTRCD

Traditional biomarkers such as troponin I and BNP, while valuable indicators of myocardial injury, lack the specificity for chemotherapy-related cardiotoxicity due to their elevation in various other pathological conditions. This limitation has driven efforts to identify novel biomarkers that can offer more precise and early detection of myocardial damage. *MicroRNAs (miRNAs)* have emerged as promising candidates for monitoring CTRCD. These small, non-coding RNA molecules regulate gene expression by interacting with mRNA [192,193]. Sánchez-Sánchez et al. [194] reported that miR-4732-3p, an miRNA with cardioprotective properties, was under-expressed in blood samples from breast cancer patients treated with anthracyclines. Additionally, other miRNAs, including miR-1, miR-21, miR-30, miR-34, and miR-133, have been shown to be significantly elevated in patients undergoing chemotherapy, particularly after treatment with trastuzumab. These findings highlight the potential of miRNAs as early and reliable biomarkers for trastuzumab-induced cardiotoxicity, allowing for timely intervention [195].

*Myeloperoxidase (MPO),* an enzyme released by neutrophils during inflammatory processes, has also been proposed as a biomarker for cardiotoxicity. Lakhani et al. found that MPO levels were markedly higher in breast cancer patients treated with doxorubicin, particularly at 3 and 6 months post-treatment, compared to healthy controls [196,197]. Similarly, inflammatory markers such as *interleukins (IL-10, IL-1β, IL-6)* have been associated with chemotherapy-induced cardiotoxicity. Alves et al. observed that elevated IL-10 levels in patients undergoing doxorubicin treatment correlated with NT-pro-BNP levels [198]. However, the use of inflammatory biomarkers in identifying cardiotoxicity is complex, as they can be influenced by the overall inflammation from the underlying cancer. It should be noted that chronic inflammation plays a crucial role in the initiation and progression of cancer, as inflammatory molecules disrupt cell signaling, promoting cancer initiation, invasion, and metastasis [129,130]. Therefore, there may be an overlap between the inflammation caused by cancer itself and that induced by cancer therapies. Elevated levels of inflammatory markers, such as cytokines and metalloproteinases, can be seen before anthracycline treatment in cancer patients, correlating with early signs of myocardial dysfunction [11,129,130]. However, results on changes in biomarker levels after anthracycline therapy are mixed. Further large-scale studies are needed to refine clinical biomarkers specific to CTRCD. *The neutrophil-to-lymphocyte ratio (NLR)* has also been identified as a predictor of cardiotoxicity risk in patients receiving anthracyclines. Baruch et al. demonstrated that patients with an NLR ≥2.58 were more likely to experience reductions in GLS during chemotherapy. Moreover, chemotherapy regimens, particularly those involving adriamycin and cyclophosphamide, have been linked to altered metabolic profiles, increasing cardiovascular risk factors such as total cholesterol and glucose levels [199]. Metabolic changes further contribute to cardiotoxicity risk. Asnani et al. observed reduced *citric acid levels* in HER2+ breast cancer patients who developed cardiotoxicity, with these reductions correlating with LVEF depression. This suggests that citric acid levels may serve as an early indicator of CTRCD [200].

In summary, recent advances in identifying novel biomarkers, including miRNAs, inflammatory markers, and metabolic profiles, offer promising opportunities for the early and accurate detection of chemotherapy-induced cardiotoxicity. These biomarkers could pave the way for more accessible and cost-effective diagnostic strategies, enabling earlier intervention and improved outcomes for at-risk patients.

### 8.4. Imaging Tecniques for Early Detection of CTRCD

*PET/CT scans*, which provide a combination of anatomical and metabolic insights, have emerged as a valuable diagnostic method. In oncology, the glucose analogue ^18^F-FDG is commonly utilized for PET/CT imaging. During periods of significant stress, cardiomyocytes may increase glycolytic activity. Elevated myocardial ^18^F-FDG uptake observed through PET/CT, indicating inflammation, could serve as an early indicator of cardiomyocyte alterations preceding cardiac dysfunction [201]. New PET and SPECT tracers are being developed to target specific CTRCD mechanisms like apoptosis and myocardial necrosis.

### 8.5. Gene Therapy in Addressing and Reversing CTRCD

Gene therapy represents a promising frontier in the quest to counteract CTRCD. Among emerging treatments with potential anti-cardiac remodeling effects, gene therapy offers the prospect of a durable and effective method for myocardial protection. Recent clinical trials investigating gene therapy for CTRCD have focused on mitigating the impact of autophagy, a critical process implicated in doxorubicin-induced HF. Excessive autophagy has been linked to cardiomyocyte loss, with studies demonstrating the overexpression of autophagic regulatory proteins, such as LC3-II, during doxorubicin treatment [202,203]. Xiaofan Sun et al. [204] explored an innovative gene therapy targeting autophagy. Their study revealed that genetic inhibition of Atg7, a protein encoded by the ATG gene and central to autophagy regulation, could slow the progression of doxorubicin-induced cardiotoxicity. Using a mouse model, Atg7 deletion was validated through Western blot analysis of myocardial tissue following tamoxifen administration. Both wild-type and Atg7-knockout mice were then subjected to weekly doxorubicin injections for four weeks. The wild-type mice exhibited a marked reduction in LVEF, which fell to approximately 40%, whereas the Atg7-knockout mice preserved systolic function with an LVEF of around 55%. Although the LVEF values in the knockout mice remained below those of the placebo-treated controls, the extent of cardiac damage was significantly reduced. Histological analyses further corroborated these findings, showing diminished autophagosome and vacuole formation in the cardiomyocytes of the Atg7-deleted animals compared to the wild-type mice, underscoring the role of autophagy in cardiotoxicity. The study also examined the effects of SAR405, an inhibitor of VPS34—a protein essential for autophagic complex formation—used alone and in combination with dexrazoxane, a chelating agent with mixed cardioprotective effects. Treatment with SAR405 reduced LC3 levels, another key autophagy regulator, in cardiac cells. When combined with dexrazoxane, SAR405 further mitigated LVEF decline, with some cases showing values approaching those of the placebo-treated controls [202,203].

In summary, targeting autophagy through gene therapy offers a compelling strategy to alleviate doxorubicin-induced cardiotoxicity. Continued research into these approaches could lead to novel therapies that improve the cardiac outcomes of cancer patients undergoing chemotherapy.

**Figure 2:**
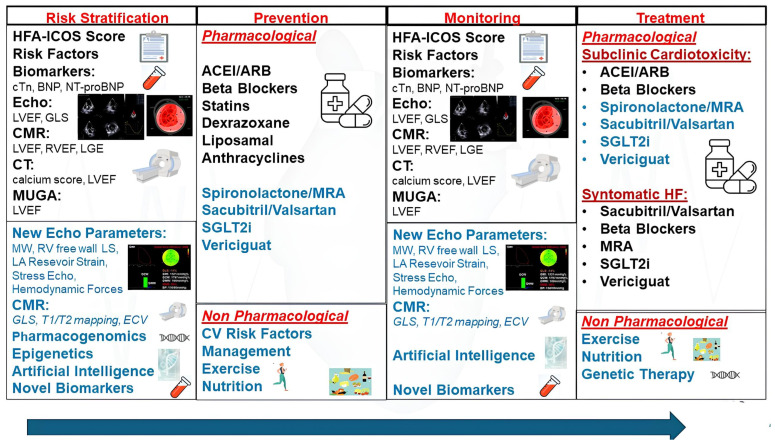
Schematic illustration of the phases of risk stratification, primary prevention, monitoring, and treatment of CTRCD. 1—Risk stratification phase: Risk stratification using the HFA-ICOS score, which takes into account the history of cardiovascular disease, baseline biomarkers, age, cardiovascular risk factors, prior exposure to cardiotoxic therapies or radiotherapy, lifestyle risk factors (smoking and obesity), baseline LVEF, and/or LV structural alterations. Echocardiography is the first-line method for evaluating LV dysfunction. Three-dimensional echocardiography is the preferred method for evaluating LVEF, while speckle tracking is employed for GLS, an indicator of subclinical systolic dysfunction. When acoustic windows are poor, CMR is recommended as the gold standard method for assessing volumes and LVEF, allowing tissue characterization too. Alternatively, if CMR is unavailable, MUGA or CT can be used to assess LVEF. 2—Primary prevention phase: Once high- and very-high-risk patients are identified, primary prevention treatment should be initiated, including pharmacological measures (e.g., ACE inhibitors/ARBs, beta-blockers, and statins) and non-pharmacological approaches (e.g., physical exercise and nutrition). 3—Monitoring phase: The risk of cardiotoxicity is dynamic and must be evaluated before, during, and after treatment, as outlined in baseline risk stratification (prior to therapy initiation). 4—Treatment phase: If a patient develops CTRCD, treatment should be tailored according to its severity, symptomatology, and the type of chemotherapy that caused it. In general (see text for more details), subclinical cardiotoxicity is managed with ACE inhibitors/ARBs and beta-blockers, while HF therapy follows standard HF guidelines. In black: methods, parameters, and treatments recommended by the 2022 ESC cardio-oncology guidelines; in blue: methods and treatments not yet recommended but supported by scientific evidence in the literature. ACEI, ACE inhibitors; ARB, angiotensin receptor blockers; CMR, cardiac magnetic resonance; CT, cardiac computed tomography; CV, cardiovascular; EF, ejection fraction; ECV, extracellular volume; GLS, global longitudinal strain; HFA-ICOS, Heart Failure Association/International Cardio-Oncology Society; LA, left atrial; LS, longitudinal strain; LV, left ventricular; MRA, mineralocorticoid receptor antagonists; MUGA, Multigated acquisition nuclear imaging; MW, myocardial work; RV, right ventricular; SGLT2i, sodium-glucose co-transporter type 2 inhibitor.

Figure 2 schematically illustrates the phases of risk stratification, primary prevention, monitoring, and treatment of CTRCD.

## 9. Conclusions

This review has highlighted the importance of early detection through advanced imaging and biomarkers, along with the role of pharmacological interventions and lifestyle modifications in mitigating CTRCD. We emphasize that the pharmacological arsenal for the prevention and treatment of CTRCD has expanded, now including established agents such as ACEI/ARBs, beta-blockers, and statins, as well as promising new therapies like ARNI, SGLT2i, and vericiguat. In addition, lifestyle modifications, including physical exercise and nutritional guidance, play a crucial supporting role in cardioprotection.

However, despite these advances, significant challenges remain. Understanding the mechanisms of toxicity associated with different cancer therapies could help to identify new therapeutic targets and optimize existing interventions. In this regard, the pursuit of personalized medicine through emerging technologies such as genomics, artificial intelligence, new biomarkers, and gene therapy offers exciting prospects for improving the prevention and treatment of CTRCD.

Finally, a multidisciplinary approach involving cardiologists, oncologists, and other healthcare professionals is essential for optimal prevention, monitoring, and treatment of CTRCD, ensuring that cancer patients not only survive the disease but also maintain a good quality of life.

## Figures and Tables

**Table 1:** Summary of the main antitumor therapies, their oncological indications, possible mechanisms of cardiotoxicity, and the frequency of left ventricular systolic dysfunction.

Treatments	Therapeutic Indications	Mechanisms of Cardiotoxicity	CRTCD/LV Systolic Dysfunction	References
**Anthracyclines** Doxorubicin Daunorubicin Epirubicin Idarubicin	Breast cancer, Gastric tumor, Leukemias, Lymphomas, Lung cancer, Ovarian tumor, Sarcomas	**↑** Toxic ROS and **↑** Oxidative stress ↓ Endogenous antioxidant enzyme Iron complex accumulation Topoisomerase IIb inhibition Mitocondrial dysfunction DNA damage Cellular apoptosis Dysregulation of calcium homeostatis	**+++**	[2,4,9,11,12,13,14]
**HER2-targeted therapies** Trastuzumab Pertuzumab	Breast cancer, Gastric cancer, Esophageal cancer	Direct blockage to HER2 protective effect from cardiotoxin Inibition of Neurugulin 1 **↑** Oxidative stress	**+++**	[2,4,9,11,12,13,14]
**EGFR/HER2** Lapatinib Osimertinib	Breast cancer, NSCLC	**↑** Oxidative stress	**++**	[3,9,14]
**Antimetabolities** Fluorouracil (5-FU) Capecitabina	Breast cancer, Colorectal cancer, Pancreatic cancer	Endothelial cell damage Coronary artery spasm Intensified sympathetic nervous system activation Potential direct toxic impact on heart muscle cells	**+**	[9,13,14]
**VEGF Receptor Monoclonal Antibodies** Bevacizumab	Colorectal cancer, Glioblastoma, Breast cancer, NSCLC, RCC	**↑** Oxidative stress Mitocondrial dysfunction	**++**	[3,9,13,14]
**VEGF Tyrosine Kinase Inhibitors (TKI)** Axitinib Cabozantinib Lenvatinib Pazopanib Regorafenib Sorafenib Sunitinib Vandetanib	RCC RCC, HCC-differentiated thyroid cancer, HCC, Endometrial cancer, RCC RCC, Soft tissue sarcoma, Colorectal cancers, RCC, HCC, GIST Pancreatic Neuroendocrine tumors, Melanoma, GIST, RCC Medullary thyroid cancer, Breast/lung cancers, CML, CEL, ALL, Mesothelioma	**↑** Oxidative stress Mitocondrial dysfunction	**++** **+** **++** **+** **+** **++** **+** **+**	[3,9,13,14]
**BCR-ABL Inhibitors** Imatinib Nilotinib Dasatinib Bosutinib Ponatinib	GIST, CML, ALL GIST, CML CML, ALL CML CML, ALL	Severe mitochondrial impairment Reduction in ATP levels Inhibition of ABL and/or ARG leads to altered cardiomyocyte function	**+** **-** **++** **++** **++**	[3,9,14]
**Bruton TKI** Ibrutinib Acalabrutinib	CLL, Mantle cell lymphoma, Waldenström macroglobulinemia, Marginal zone lymphomas		**+**	[3,9,13,14]
**Proteasome Inhibtors (PI)** Carfilzomib Bortezomib	Multiple myeloma	**↑** Oxidative stress	**++** **+**	[3,9,13,14]
**Immune checkpoint inhibitors (ICI)** **CTLA-4 inhibitor** Ipilimumab **PD-1 inhibitors** Nivolumab Pembrolizumab	Colorectal cancer, HCC, Melanoma, NSCLC, RCC As above, plus Esophageal cancer, Head and neck Hodgkin lymphoma, Small-cell lung cancer Urothelial carcinoma As above, plus Cervical cancer, Cutaneous squamous cell Carcinoma, Endometrial carcinoma, Gastric cancer, Merkel cell carcinoma, Primary mediastinal large, B-cell Lymphoma	Unknown Generation of autoantibodies and production of proinflammatory cytokines	**++**	[2,3,9,14]
**CAR T-Cell Therapy**	ALL, Diffuse large B-cell lymphoma	Cytokine release	**++**	[3,9,14]
**Alkylanting Agents** Cyclophosphamide Ifosfamide Melphalan Platinum Dugs Cisplatin Carboplatin Oxaliplatin	Leukemias, Lymphomas, Various solid tumors CML, Germ cell tumors, Ovarian cancer, Lymphomas, Head/neck tumors, Lung cancer, Sarcomas	Endothelial capillary damage Anaphylaxis and hypersensitivity reaction Endothelial injury/ apoptosis	**+** **+** **++** **+**	[3,9,13,14]
**Taxans (Microtubule Inhibitors)** Paclitaxel Docetaxel	Breast cancer, Ovarian cancer, NSCLC, Kaposi’s sarcoma, Prostate cancer, Gastric Adenocarcinoma	Reduced calcium in cardiomyocytes, resulting in decreased contraction	**++**	[3,9,13,14]
**Hormonal Therapy** Apalutamide Bicalutamide Darolutamide Nilutamide	Prostate cancer	Induction of a metabolic syndrome condition (dyslipidemia, increased body fat, and insulin resistance) Promotion of atherosclerosis development	**++**	[14]
**Immunomodulatory Drugs** Lenalidomide Pomalidomide Thalidomide	Multiple myeloma, MDS, Mantle cell lymphoma Multiple myeloma, Kaposi sarcoma Multiple myeloma	Unknown	**++** **+** **++**	[9,14]
**MEK Inhibitors** Binimetinib Cobimetinib Trametinib Selumetinib	Melanoma Melanoma Melanoma, NSCLC, Anaplastic thyroid cancer Neurofibromatosis Type 1	Inhibition of MAPK signaling: **↑** Oxidative stress Cellular apostosis	**++** **+++** **++** **+++**	[3,9,14]
**BRAF (rapidly accelerated fibrosarcoma B TYPE):** Vemurafenib Dabrafenib	Melanome NSCLC	Inhibition of MAPK signaling: **↑** Oxidative stress Cellular apostosis	**+++**	[3,9,14]
**Haematopoietic Stem Cell Transplant (HSCT)**	Hematological malignancies		**+**	[3,9,14]
**RT**	Breast, Lung, Oesophagus, Thyroid, Prostate cancer, Mediastinal lymphoma, Head and neck tumors	Damages mitochondria Activates NADPH oxidase to generate ROS, which in turn exacerbate mitochondrial damage	**++**	[2,9,13,15]

+: uncommon and rare or <1%; ++: common or 1–10%; +++: frequent or >10%. ↑: increased; ↓: decreased; ALL, acute lymphoblastic leukemia; CEL, chronic eosinophilic leukemia; CML, chronic myelogenous leukemia; GIST, gastrointestinal stroma tumor; HCC, hepatocellular carcinoma; MDS, myelodysplastic syndrome; NSCLC, non-small-cell lung carcinoma; RCC, renal cell carcinoma; ROS, reactive oxygen species.

**Table 2:** Clinical trials evaluating pharmacological cardioprotective strategies for the primary prevention of CTRCD.

Study	Chemotherapy	Cancer Type	Intervention	Follow-Up	N. Patients	Primary Endpoint and Results	Secondary Endpoint and Results
CECCY Avilla et al. [98] 2018	Anthracyclines	Breast Cancer	Carvedilol vs. placebo	6 months	192	Early onset drop in LVEF ≥10% by echo Carvedilol had no impact	Changes in TnI, BNP, and diastolic dysfunction Significant reduction in TnI and diastolic dysfunction
Tashakory et al. [96] 2016	Anthracyclines	Brest Cancer	Carvedilol vs. placebo	1 week	70	Early onset drop in LVEF ≥10% and drop in GLS by echo Carvedilol had no impact	
SAFE Livi et al. [105] 2021	Anthracyclines ± Trastuzumab	Breast Cancer	Bisoprolol vs. placebo Ramipril vs. placebo	12 months	174	Early onset drop in LVEF ≥10% and drop in GLS by echo Significant minor reduction in LVEF and GLS in bisoprolol group vs. placebo group No effect of Ramipril	
ICOS-ONE Cardinale et al. [82] 2018	Anthracyclines	Breast cancer, Sarcoma, Hematological malignancies	Enalapril primary prevention vs. troponin-guided prevention with enalapril	12 months	273	Incidence of troponin elevation No differences between primary prevention with enalapril- and troponin-guided prevention	
PRADA Gulati et al. [88] 2016	Anthracyclines ± Trastuzumab	Breast cancer	Candesartan, Metoprolol succinate, or matching placebo	10–61 weeks	126	Change in LVEF > 5% by CMR Metoprolol had no effect Candesartan protected against early LVEF decline vs. Metoprolol and placebo group	
Boekhout et al. [131] 2016	Trastuzumab	Breast Cancer	Candesartan vs. placebo	26 weeks	206	Decline in LVEF > 15% or a decrease in the LVEF <45% Candesartan had no effect	
Akpek et al. [103] 2015	Anthracyclines	Breast Cancer	Spironolactone vs. placebo	3 weeks	83	Change in LVEF ≥ 10% by echo and increase in TnI LVEF decrease and increase in TnI significantly lower with Spironolactone; no impairment in diastolic function	
Acar et al. [123] 2011	Anthracyclines	Lymphoma, MM, Leukemia	Atorvastatin vs. placebo	6 months	40	Absolute change in LVEF by echo Atorvastatin was effective in maintaining LVEF vs. placebo	
OVERCOME Bosch et al. [101] 2013	Anthracyclines	Hematological malignances	Carvedilol + enalapril	6 months	90	Absolute change in LVEF by echo and CMR Both carvedilol and enalapril prevented LVEF drop by echo and by CMR	Patients in carvedilol + enalapril group had lower incidence of combined event of death or HF death, HF or a final LVEF < 45%
Janbabai et al. [132] 2017	Anthracyclines	Breast cancer, Hematological malignances	Enalapril	6 months	69	Absolute change in LVEF, E/e’ ratio, TnI elevation Enalapril prevented: decline in LVEF, elevation of E/e’ ratio, and elevation of TnI	
MANTICORE Tashakori et al. [96] 2016	Trastuzumab	Breast cancer	Bisoprolol, perindopril, or placebo	12 months	94	LV remodeling: change in LVEDVi by CMR Bisoprolol and perindopril had no effect	
STOPCA Neilan et al. [124] 2023	Anthracyclines	Lymphoma	Atorvastatin vs. placebo	12 months	300	Proportion of participants with an absolute decline in LVEF of ≥10% from that prior to chemotherapy to a final value of <55% Atorvastatin reduced the incidence of cardiac dysfunction	Proportion of participants with an absolute decline in LVEF of ≥5% from that prior to chemotherapy to a final value of <55% Atorvastatin reduced the incidence of cardiac dysfunction
PREVENT Hundley et al. [133] 2022	Anthracyclines	Breast cancer, Lymphoma	Atorvastatin vs. placebo	24 months	279	Absolute change in LVEF by CMR No difference between the two groups	
CARDIAC-CARE Henriksen et al. [104] 2023	Anthracyclines	Breast cancer, Lymphoma	Carvedilol + candesartan	6 months	175	Adjusted change in LVEF by CMR No effect in patients with high-risk on-treatment cardiac troponin I concentrations	

CMR, cardiac magnetic resonance; GLS, global longitudinal strain; LVEDVi, left ventricular end diastolic volume index; LVEF, left ventricular ejection fraction; MM, multiple myeloma; TnI, troponin I.

**Table 3:** Meta-analyses testing pharmacological cardioprotective strategies for the primary prevention of CTRCD.

Meta-Analyses	Chemotherapy	Number of Patients	Intervention	Results
Huang et al. [100] 6 RCTs 2019	Anthracyclines	495	Beta-blockers (carvedilol) vs. placebo	LVEF was not significantly distinct between the two groups (MD: 1.74; 95% CI −0.18 to 3.66; *p* = 0.08) Clinically overt cardiotoxicity was lower in the carvedilol group (Peto OR, 0.42; 95% CI 0.20–0.89; *p* = 0.02).
Li et al. [134] 22 RCTs 2020	Anthracyclines, Trastuzumab, Cyclophosphamide, Taxanes, Platinum agents, 5-FU, and others	1916	ACEI vs. placebo ACEI vs. control Statins vs. control Beta-blockers vs. placebo	Significant reduction in decline in LVEF: Spironolactone (MD: 12.77, 95% IC: 1.76–23.79) Candesartan and carvedilol (MD 12.40, 95% IC: 0.99–23.81) Enalapril (MD: 7.35, 95% IC: 1.16–13.54) Statin (MD 8.36, 95% IC: 0.36–16.36) compared with placebo
Fang et al. [135] 9 RCTs 2021	Anthracyclines ± Trastuzumab	1095	ACEI/ARB vs. placebo	Significantly lower reduction in LVEF in ACEI/ARB receivers (MD: 4.24%, 95% IC: 1.53–6.95; *p* = 0.002) No significant reduction in the risk of cardiotoxicity events (RR: 0.63, *p* = 0.22) Significant increase in hypotension (RR: 3.94, *p* = 0.008)
Caspani et al. [136] 12 RCTs 2021	Anthracyclines	2177	RAAS blockers, beta-blockers, and aldosterone antagonists	Significantly lower reduction in LVEF in the intervention arm (MD: 3.57, 95% CI 1.04, 6.09) No significant reduction in HF (OR 0.31, 95% CI 0.06, 1.59; 5 studies) in cardioprotected arm No significant increase in hypotension (OR 3.91, 95% CI 0.42, 36.46, 3 studies) in cardioprotected arm
Attar et al. [97] 17 RCTs 2022	Anthracyclines	1291	Beta-blockers (carvedilol, bisoprolol, Nebivolol, and Metoprolol)	Significantly lower reduction in LVEF in beta-blocker receivers (MD: 3.44%, 95% CI: 1.41–5.46, *p* = 0.001, I^2^ = 94.0%) Among the 8 studies reporting the incidence of CTRCD, there was no significant reduction in CTRCD incidence in beta-blocker receivers (RR: 0.76; 95% CI: 0.53–1.09; I^2^ = 24.4%; *p* = 0.235)
Keshavarzian et al. [107] 728 studies 2023	Anthracyclines	2674	Dexrazoxane, beta-blockers, ACEI	Increase in LVEF in the intervention group by 0.40 after 6 months (SMD: 0.40, 95% CI 0.27 to 0.54; *p* <0.05)
Titus et al. [126] A total of 3 observational studies, 4 RCTs 2023	Anthracyclines	2511	Statins	Significantly lower incidence of cardiotoxicity in patient who used statins compared to non-users (OR 0.46, 95% CI 0.33–0.63; I^2^: 0%) No significant difference in the decline in LVEF from the baseline (MD: 4.15, 95% CI Ȓ0.69 to 8.99, I^2^: 97%)
Gao et al. [137] 15 RCTs 2023	Anthracyclines ± Trastuzumab	1977	ACEI/ARB and/or beta-blockers vs. placebo	Significant reduction in decline in LVEF in ACEI/ARB and beta-blocker groups (*X*^2^*:* 184.75, I^2^: 88.6%; *p* = 0.000; MD: 0.556, 95% IC: 0.299–0.813)

ACEI, ACE inhibitors; ARB, angiotensin receptor blockers; CTRCD, cancer-therapy-related cardiac dysfunction; HF, heart failure; LVEF, left ventricular ejection fraction; RAAS, renin–angiotensin–aldosterone system; RCT, randomized controlled trial.
